# Effects of the Mixing Method of Expanded Graphite on Thermal, Electrical, and Water Transport Properties of Thermosetting Nanocomposites

**DOI:** 10.3390/polym17202759

**Published:** 2025-10-15

**Authors:** Raffaele Longo, Elisa Calabrese, Francesca Aliberti, Luigi Vertuccio, Giorgia De Piano, Roberto Pantani, Marialuigia Raimondo, Liberata Guadagno

**Affiliations:** 1 Department of Industrial Engineering, University of Salerno, Via Giovanni Paolo II, 132, 84084 Fisciano, Italy; elicalabrese@unisa.it (E.C.); faliberti@unisa.it (F.A.); gdepiano@unisa.it (G.D.P.); rpantani@unisa.it (R.P.); mraimondo@unisa.it (M.R.); 2 Department of Engineering, University of Campania “Luigi Vanvitelli”, Via Roma 29, 81031 Aversa, Italy; luigi.vertuccio@unicampania.it

**Keywords:** expanded graphite, epoxy resin, nanocomposites, mixing

## Abstract

The present research aims to investigate the impact of various mixing techniques (centrifugal planetary mixing, ultrasonication, and high-temperature magnetic stirring) on the properties of nanocomposite epoxy resins using expanded graphite particles. Differential scanning calorimetry reveals that the curing behavior and glass transition temperature are influenced by the selected method, indicating that a suitable choice allows increasing curing degree (C.D.) and glass transition temperature up to 10% and 12%, respectively. Morphological analysis performed via Scanning Electron Microscopy and Tunneling Atomic Force Microscopy offers detailed insights into the dispersion characteristics of fillers within polymer matrices, which sensitively affect the properties of the materials. The electrical conductivity values vary by more than five orders of magnitude among the various mixing methods. Centrifugal mixing leads to a decrease in the equilibrium concentration of water (C_eq_) by up to 23% compared to that of the unfilled matrix, thanks to the chemical interactions that occur between the graphitic particles and the epoxy matrix (detectable via Fourier Transform Infrared Spectroscopy). Such a reduction is strongly desired in strategic fields such as the transport sector. The analysis of the obtained results suggests choosing the dispersion method of the filler in the matrix by considering the required performance for the specific planned application.

## 1. Introduction

The inclusion of fillers inside polymeric matrices is a method widely explored to enhance or impart several properties to the material [1,2,3,4]. By choosing the filler–matrix combination wisely, it is possible to develop materials with characteristics that are sensitively different from the starting matrix [5,6,7,8,9]. For example, it is possible to increase the electrical conductivity of polymeric materials (generally insulating) by several orders of magnitude by including metallic or carbonaceous fillers (e.g., carbon nanotubes, graphene, etc.) inside the matrix [10,11,12,13]. In this case, starting from an electrically insulating polymeric matrix, it is possible to realize a semiconductive composite, tuning the material’s electrical properties depending on the application field [14].

The nanofillers are also widely used to improve the barrier properties of the materials to the external environment [15,16]. The fields of application are extensive, ranging from food packaging, in which the nanofillers help to decrease the values of thermodynamic and kinetic parameters, characteristics of the permeability of the nanocomposites, to the transport sector, in which it is often required to reduce the sensitivity of the system to the humidity [17,18,19]. It is well-known that water sorption causes plasticization of the material, generally causing a decrease in the thermomechanical and mechanical performance of the material [20,21,22]. For this reason, the development of materials with enhanced water barrier properties, applicable in the transport sector (e.g., epoxy resins), is strongly requested [23].

Analysis of the existing literature on the development of these composite systems reveals a significant variability in performance metrics, which are influenced by the specific types of matrices and fillers employed [24,25,26]. For example, it is widely accepted that thermosetting matrices loaded with electrically conductive nanoparticles generally present a lower electrical percolation threshold than thermoplastic polymers loaded with the same nanofiller [27]. Furthermore, the selection of filler type (aspect ratio, surface areas, functionalization, etc.) can significantly influence the physical and mechanical properties of composite materials, primarily by affecting the dispersion of the filler and the filler–matrix interaction [28,29]. Moreover, the processing temperature can also affect the performance of the material, causing phenomena of thermal aggregation of the filler in the fluid phase (i.e., in the extruder for the thermoplastics, in the reactive mixture for the thermosettings) [30,31].

In the literature, a few papers have evaluated the effect of dispersion on the properties of the materials. Chen et al. [32] assessed the impact of the dispersion methods on the thermomechanical and tribological properties of epoxy resin filled with carbon nanotubes; Prolongo et al. [33] investigated the effect of two different mixing methods of carbon nanofibers in epoxy composites, highlighting differences in the storage modulus. Shen et al. [34] analyzed the differences in the mechanical properties of samples produced via centrifugal planetary mixing and three-roll milling at low concentration (below 1% in weight).

The current study investigates the influence of various nanofiller mixing techniques within a model epoxy matrix, with a detailed assessment of how filler dispersion impacts the resulting material properties. For this analysis, a filler content of 7% by weight was selected, as this concentration is beyond the Electrical Percolation Threshold (EPT) for all the mixing methods. The authors decided to define the EPT of the mixture by using the centrifugal planetary mixing, given the high industrial scalability of this method.

The samples were produced using various mixing methods, which were found to influence the distribution and connectivity of the filler within the matrix. Understanding the impact of mixing methods on different properties is crucial for optimizing electrical conductivity, thermal properties (such as curing degree and consequently mechanical performance), and transport properties, especially in applications requiring rigorous control of parameters correlated to these properties. Morphological analysis employing Scanning Electron Microscopy (SEM) and tunneling atomic force microscopy (TUNA) was performed to investigate the distribution of nanofillers, hence the formation of electrical conduction pathways, and the eventual presence of agglomerates within the epoxy composite matrix. Fourier Transform Infrared Spectroscopy (FT-IR) was employed to elucidate the interaction mechanisms between the graphitic nanofiller and the epoxy matrix, providing insights into their interfacial chemistry and bonding characteristics. Structural analysis using X-Ray Diffraction (XRD) was performed to determine the degree of exfoliation of the expanded graphite before and after mixing. The electrical and barrier properties of the epoxy composites were evaluated to understand how variations in nanofiller dispersion influence the overall material performance.

## 2. Materials and Methods

### 2.1. Resin Development

Epoxy diglycidyl-ether bisphenol-A (DGEBA) and 4,4′-diaminodiphenyl sulfone (DDS) were obtained from Sigma-Aldrich. Expanded graphite (EG) ABG1045 was obtained by Superior Graphite (550 W. Van Buren St., Suite 300, Chicago, IL, USA).

The epoxy resin is prepared by including the expanded graphite (EG) in the precursor. In the present research paper, three different mixing methods have been used for the preparation of the epoxy resin:

- Ultrasonication: The epoxy precursor and EG, at the defined weight percentage over the total mixture, were mixed using an ultrasonication treatment for 20 min (Hielscher model UP200S-24 kHz high power ultrasonic probe, manufactured by Hielscher Ultrasonics GmbH Oderstr. 53, 14513 Teltow, Germany). The ultrasonic parameters were as follows: 200 W, 100% amplitude, pulse 50%.
- High-Temperature Mixing: The epoxy precursor and EG, at the defined weight percentage over the total mixture, were mixed at high temperature (120 °C for 20 min) under magnetic stirring (300 rpm).
- Centrifugal Planetary Mixing: The epoxy precursor and EG, at the defined weight percentage over the total mixture, were mixed using a centrifugal planetary mixer (THINKY ARE-250, manufactured by THINKY Corporation, Tokyo, Japan), for 1 min at 2000 rpm (mixing) and 2 min at 2000 rpm (defoaming).

After the inclusion of EG into the epoxy precursor, DDS was dissolved in the DGEBA-EG mixture at 120 °C for 75 min under magnetic stirring (300 rpm) and then degassed under vacuum at 80 °C for 2 h. The epoxy mixture was then poured into a metallic mold, and it was cured for 1 h at 130 °C and 3 h at 200 °C.

An overview of the samples produced is reported in Table 1.

### 2.2. Epoxy Resin Characterization

The methods used to characterize the epoxy resin are described in Section S1 of the Supplementary Materials.

## 3. Results

### 3.1. Thermal Stability

**Figure 1:**
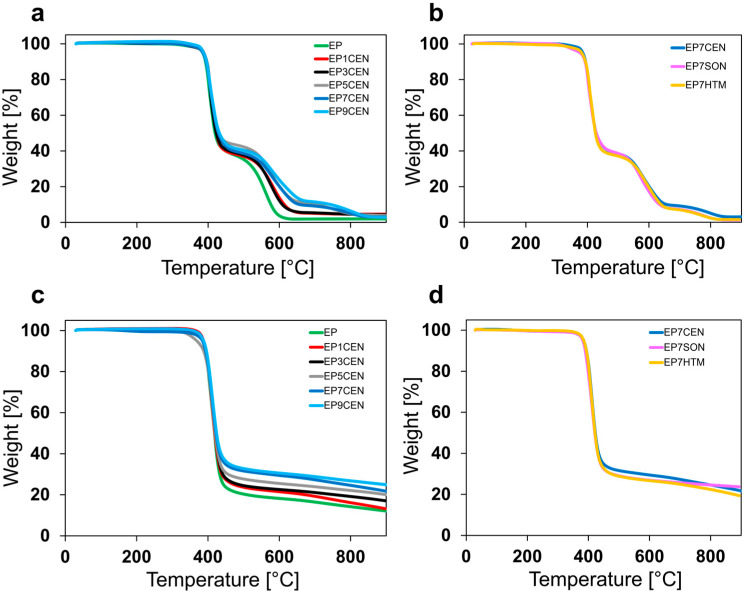
TGA in airflow of the epoxy samples at various EG concentrations (**a**) and with different mixing methods (**b**). TGA in nitrogen flow of the epoxy samples at various EG concentrations (**c**) and with varying methods of mixing (**d**).

The epoxy samples reported in Table 1 have been characterized to evaluate their thermal stability in air and N_2_. The thermograms of TGA are reported in Figure 1.

From the analysis of the results, it is evident that the degradation of the EP sample occurs in two main stages. The first stage, common to both TGA performed under N_2_ flow and under air flow, is linked to thermolysis; the increase in temperature causes the breakage of the crosslinked network, leading to the production of smaller molecules [35]. If the produced molecules are volatile enough, they leave the sample, causing a decrease in the sample’s weight. In the presence of an oxidative environment, a second step occurs at higher temperatures, where the organic materials related to the resin undergo thermo-oxidation [35]. From this analysis, it is evident that thermal degradation is closely related to both the epoxy resin and the environment. Hence, the thermal degradation is poorly affected by the EG dispersion method into the thermosetting resin, as confirmed also by the Derivative TGA (DTGA), reported in Figure S2, and by the relevant thermal parameters in Table S1 (i.e., the temperatures of the 5 wt% weight loss (T_5%_) and the 50 wt% weight loss (T_50%_)) of the Supplementary Materials Lastly, as expected, the higher EG content in the epoxy resin leads to a higher residual after exposure at high temperature, as it is possible to observe in Figure 1a,c.

### 3.2. DSC Analysis

**Figure 2:**
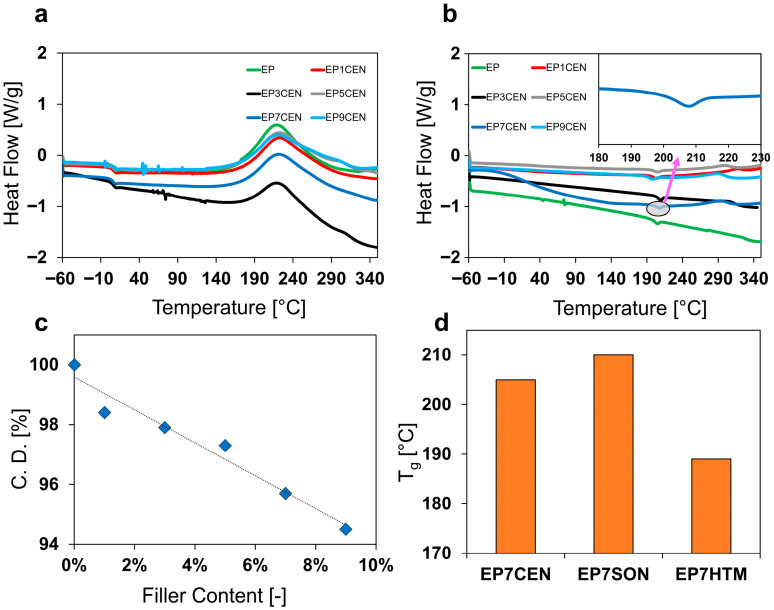
DSC analysis of the uncured mixtures (**a**) and the cured samples (**b**); influence of filler content over the curing degree for the system EPXCEN (**c**); influence of the mixing method on T_g_ (**d**).

DSC analysis of the different samples was performed to evaluate the effect of the dispersion method on the curing degree of the epoxy resin. DSC thermograms are shown below, in Figure 2. In particular, Figure 2a shows the thermograms of the uncured mixtures of the samples mixed with the centrifugal planetary (EPXCEN samples) together with the unfilled uncured sample (a), and Figure 2b shows the thermograms of the same cured samples (b). From the thermogravimetric data, the influence of the filler content on the curing degree of EPXCEN samples has been evaluated. The results are reported in Figure 2c. The influence of the mixing method on the glass transition temperature (T_g_) can be observed in the histogram of Figure 2d for all samples loaded with 7% by weight of nanofiller.

As shown in Figure 2c, the increase in filler content results in a decrease in the curing degree of the epoxy formulation, leading to the presence of a residual exothermic peak at high temperatures (above 280 °C), as observed in Figure 2b. This result is likely due to the presence of EG. In fact, the graphitic sheets at the interface between filler and matrix can act locally by separating some of the functional groups (epoxy rings and hardener), thus preventing the polymerization reactions at the interface between filler and matrix. This occurrence is rather obvious because the presence of graphitic blocks necessarily interrupts the crosslinking network. On the other hand, from a kinetic point of view, graphitic layers in the hosting matrix reduce the mobility of the epoxy resin and hinder the movements of the reactive groups [36]. Indeed, many authors found that the inclusion of graphitic particles can lead to a decrease in curing kinetics [37,38]. This hypothesis is further confirmed by the kinetic analysis performed in isothermal conditions at 160 °C and in dynamic conditions at 10 °C/min on EP and EP7CEN, by which it is evident that the inclusion of the EG into the epoxy matrix generally causes a decrease in the curing kinetics. The results are reported in Figure S3 of the Supplementary Materials.

By analyzing the T_g_ in the various thermograms of cured EP7CEN, EP7SON, and EP7HTM (see Figure 2d), it is evident that the T_g_ of the sample is significantly affected by the mixing method. In particular, EP7SON exhibits a glass transition temperature of approximately 212 °C, which is higher than that of EP7CEN (206 °C) and EP7HTM (189 °C). This difference is not correlatable only with the difference in curing degree among the three systems (C.D._EP7CEN_ = 95.8%; C.D._EP7SON_ = 98.4%; C.D._EP7HTM_ = 95.7%), which leads to a different crosslinking density and, consequently, to different values in the glass transition temperature, but also to the dispersion method. In fact, samples with a very similar curing degree, EP7CEN (C.D. = 95.8%) and EP7HTM (C.D. = 95.7%), exhibit T_g_ values of 206 and 189 °C, respectively.

The difference in terms of T_g_ among the three types of mixing methods, reported in Figure 2d (evaluated from the inflection point in the thermogram, see inset of Figure 2b), is most likely to be attributed to the different arrangement and distribution of EG within the epoxy matrix, which causes constrictions in molecular movement over different spatial ranges.

### 3.3. FT-IR Analyses

**Figure 3:**
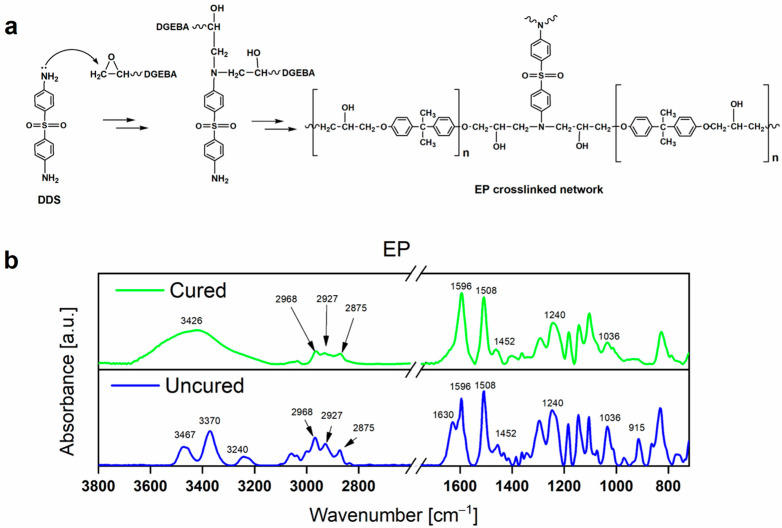
(**a**) Scheme illustrating the mechanism of the polymerization reaction. (**b**) FT-IR spectra of the uncured (see blue curve) and cured (see green curve) epoxy sample EP.

The FT-IR investigations were performed on both the cured and uncured EP samples to elucidate the polymerization mechanism, as shown in Figure 3a. According to the illustrated reactions, the nucleophilic attack of the primary amino group of the hardener (DDS) leads to the opening of the oxirane ring of the epoxy precursor (DGEBA), resulting in the formation of additional hydroxyl groups (OH) on the final EP crosslinked network. The comparison shown in Figure 3b highlights that the N-H bands belonging to the primary amino group (NH_2_) of DDS, observable in the spectrum of the uncured material (see blue curve), disappear in the spectrum of the polymerized material (see green curve), where a broad band between 3670 and 3150 cm^−1^ (centered at 3426 cm^−1^), belonging to the stretching vibrations of the OH group, appears. Furthermore, the characteristic signals of the oxirane ring stretching vibrations disappear in the cured material, while the peaks of the main skeleton of the epoxy chain, that is, C-H stretching vibrations of the CH_3_ and CH_2_ groups, C=C stretching vibrations of the benzene ring, and C-O-C stretching of alkyl-aryl ethers, remain unchanged after the curing reaction. Details of the assignments of the FT-IR signals are shown in Table 2.

**Figure 4:**
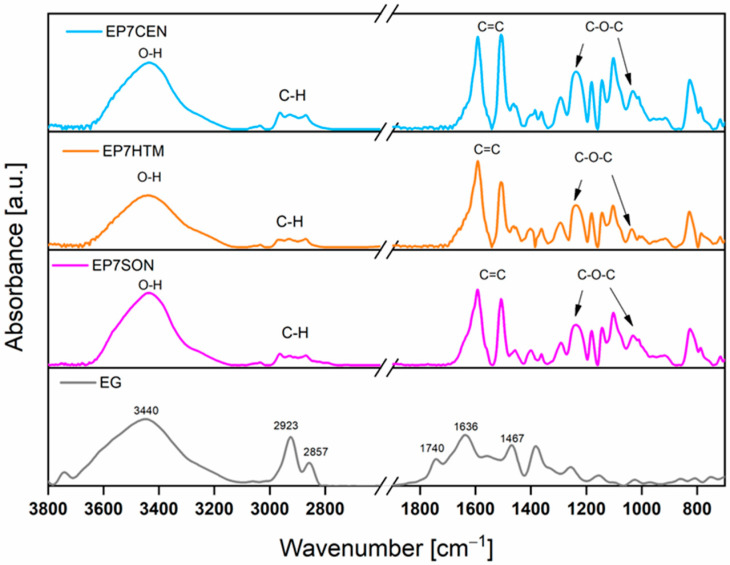
FT-IR spectra of the samples: EG (gray curve), EP7SON (magenta curve), EP7HTM (orange curve), and EP7CEN (blue curve).

**Figure 5:**
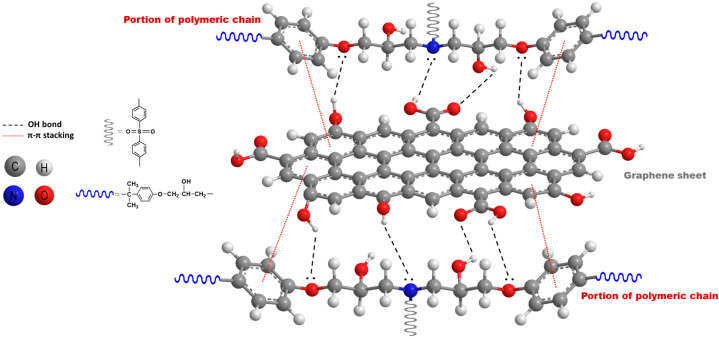
Representation of the intermolecular interactions involving the exfoliated graphite layers and the polymer chains.

FT-IR spectroscopy was used to investigate the interactions occurring between the filler and the epoxy matrix in the cured composites. Figure 4 compares the FT-IR spectrum of the nano-filler EG with those of the epoxy-based samples containing EG. The spectra of the filled samples (EP7SON, EP7HTM, and EP7CEN) are similar to those of EP (see Figure 3b). In contrast, the spectrum of EG sample displays the characteristic absorption features of EG: a broad band centered at 3440 cm^−1^, corresponding to the OH stretching vibration; the signals at 2923 and 2857 cm^−1^, belonging to the stretching bands of CH_2_; the peaks at 1636 and 1467 cm^−1^, ascribed to the skeletal vibrations of C=C; and the signal at 1740 cm^−1^, belonging to a carbonyl group. These findings indicate that EG is composed of oxidized graphite sheets, whose basal planes are predominantly functionalized with hydroxyl and carbonyl groups [14]. These groups can interact well with the hosting epoxy matrix through the formation of reversible bonding mechanisms, particularly hydrogen bonds. These interactions can be established between H-bond donor groups, such as hydroxyl groups, and H-bond acceptor groups, such as carbonyls or atoms having lone electron pairs (O and N). In addition, another kind of non-covalent reversible interaction, which can involve the nano-filler and the matrix, is the π-π stacking interaction that, together with the H-bond, can contribute to improving the nano-filler’s dispersion inside the epoxy matrix [42]. The above-mentioned interactions, which can occur between a graphene sheet and the polymeric chains, are depicted in Figure 5.

Infrared spectroscopy is an efficient method for evaluating the nature and extent of hydrogen bonding within a material, through the analysis of the hydroxyl group band profile (3650–3100 cm^−1^). In a cured epoxy resin, this signal appears as a broad band having a more or less accentuated shoulder at a lower frequency (3500–3100 cm^−1^) diagnostic for the presence of OH groups involved in H-bonds. To evaluate the effect of the EG dispersion on the H-bond interactions in the epoxy matrix, the OH bands (3700–3100 cm^−1^) of the epoxy-based cured samples were decomposed into different components using a complex fitting in which a Gaussian contribution was considered, as in previous papers [43,44].

Generally, a “free O-H band” is placed at a higher wavenumber region of the O-H stretching vibration with respect to the vibration corresponding to the O-H groups involved in hydrogen bonding. For small molecules with single signals, the “free O-H” peak is a sharp signal, and the hydroxyl groups (O-H) involved in hydrogen bonding appear as a broader, lower-frequency absorption band. These last O-H groups can sensitively sense very different chemical surroundings. For cured epoxy systems, we can detect a situation where all these signals overlap. It is worth noting that, in the resulting profile of the band, three clearly distinguishable signals are observable. In particular, a strong signal at about 3430 cm^−1^ and two shoulders, one at a higher frequency (about 3600 cm^−1^) and the other at a lower frequency (around 3250 cm^−1^) with respect to the central one, are observed. A free deconvolution was performed, considering three signals, which, after the deconvolution, appeared at frequencies very similar to those experimentally detected. The O-H bond groups sense three surrounding chemical environments, which are prevalent in determining the final profile of the O-H band.

Most likely, we can distinguish the contribution of a very small fraction of stretching vibrations related to almost “free O-H” groups and two contributions related to two different fractions of O-H groups involved in hydrogen bonding. In particular, in these last fractions, O-H groups can have a different chemical surrounding, depending on their location in the crosslinked matrix and the presence of the thin graphitic blocks, for which hydrogen bonding interactions with polymeric chains can be established due to the functional groups on EG particles.

For this reason, a deconvolution of the O-H band was performed, considering three different peaks and using a complex fitting, in which a Gaussian contribution was considered.

**Figure 6:**
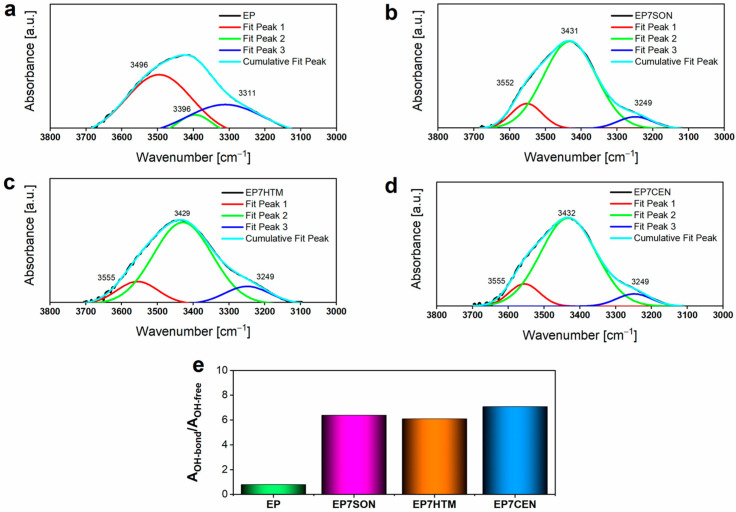
Results of the deconvolution procedure performed on the OH band for the samples: (**a**) EP, (**b**) EP7SON, (**c**) EP7HTM, (**d**) EP7CEN. (**e**) Histogram illustrating the ratios’ values A_OHbond_/A_OHfree_ for the analyzed samples.

Figure 6a–d illustrate the results of this deconvolution procedure, in which three different peaks were identified: the Peak 1 (at 3496 cm^−1^ for EP and at about 3555 cm^−1^ for the nanocomposites), attributed to non-hydrogen bonded or “free” hydroxyl groups, and the Peak 2 (at 3396 cm^−1^ for EP and around 3430 cm^−1^ for the nanocomposites) and the Peak 3 (at 3311 cm^−1^ for EP and at about 3249 cm^−1^ for the nanocomposites), which were both ascribed to OH groups involved into hydrogen bonding interactions, as previously described. The evaluation of the areas under the peaks enabled an assessment of the quantity of H-bonded OH groups (OH-bond) and of “free” OH groups (OH-free). In particular, the ratio between the area associated with the OH-bond (A_OH-bond_, obtained from the sum of the areas of Peak 2 and Peak 3) and the area ascribed to OH-free (A_OH-free_) was considered to assess the extent of H-bonding interactions. The obtained results are reported in the histogram of Figure 6e, and the data clearly show an increase in hydrogen bonds in all the samples loaded with EG, which favors the dispersion of the nano-filler inside the epoxy matrix. Furthermore, it is possible to observe that, for the nano-filled samples, the increase in the total area attributed to the OH bond is mainly due to the increase in Peak 2, highlighting the establishment of strong dynamic interactions between the polymer chains and the nano-filler [45]. Peak 3 is most likely attributed to H-bonds, due to the hydroxyl groups among polymeric chains of the matrix, as shown in Figure 6a, where the intensity of Peak 3 at 3311 cm^−1^ is higher than the intensity of the same peak in the samples loaded with the nanofiller (Figure 6b,d). The R^2^ values of the complex fitting of the curves given by the three peaks are reported in Table S2 of the Supplementary Materials section.

The results in Figure 6e clearly demonstrate that the state of the EG filler distribution in the EP7CEN sample can promote more effective interactions between the filler and the hosting matrix through H-bonding interactions.

Moreover, from a structural analysis performed via XRD, it emerges that the samples obtained with the various mixing methods slightly favor the assembling of graphene layers of the EG. Hence, the results commented on all the properties manifested by the filled samples must be attributed only to a different distribution of the thin graphitic block in the hosting epoxy matrix.

The XRD results are reported in Section S5 of the Supplementary Materials.

### 3.4. Morphological Analyses

**Figure 7:**
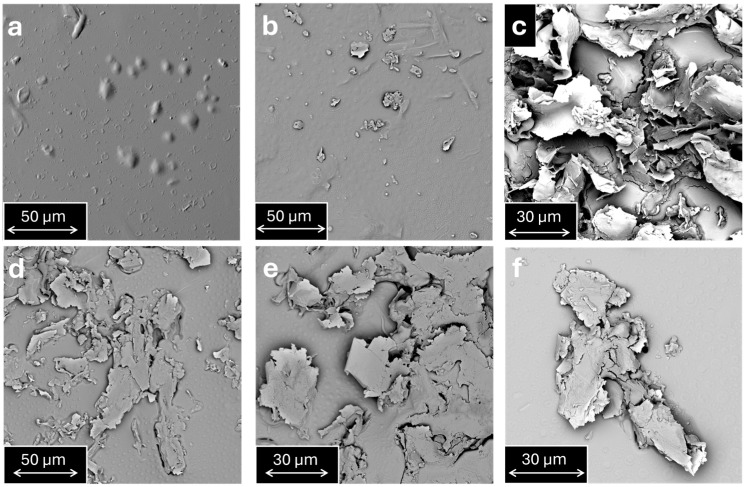
SEM analysis of EP (**a**), EP3CEN (**b**), EP9CEN (**c**), EP7CEN (**d**), EP7SON (**e**), and EP7HTM (**f**).

**Figure 8:**
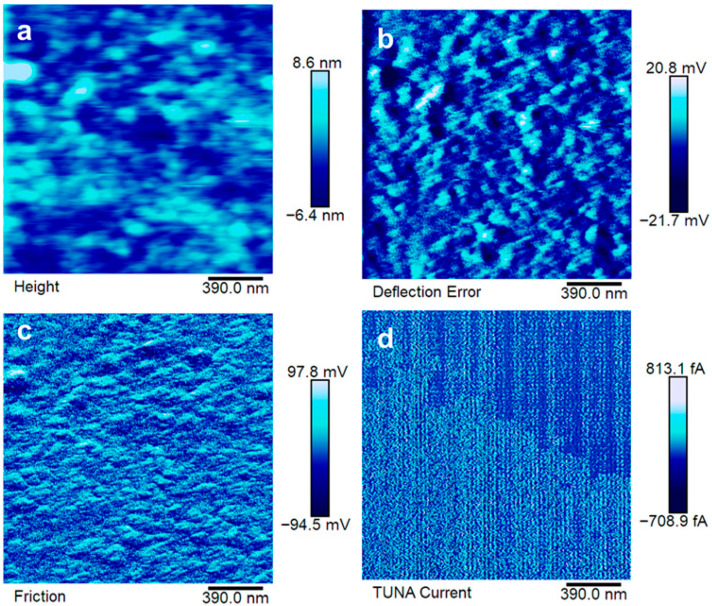
Atomic force microscopy images of EP7CEN: height image (**a**), deflection error image (**b**), friction image (**c**), TUNA current (**d**).

**Figure 9:**
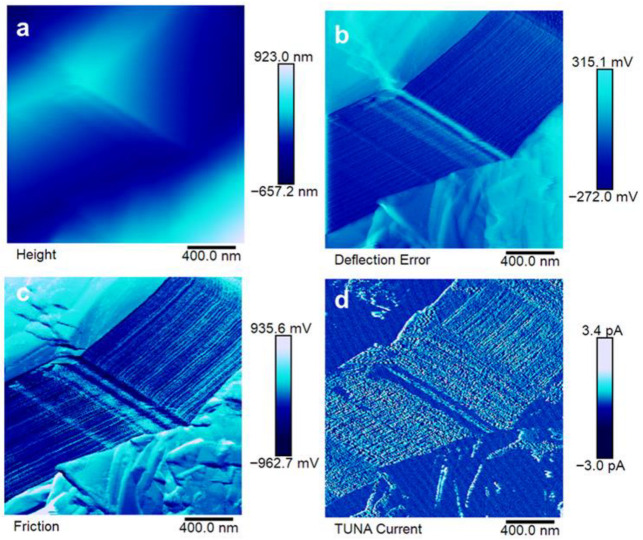
Atomic force microscopy images of EP7SON: height image (**a**), deflection error image (**b**), friction image (**c**), TUNA current (**d**).

**Figure 10:**
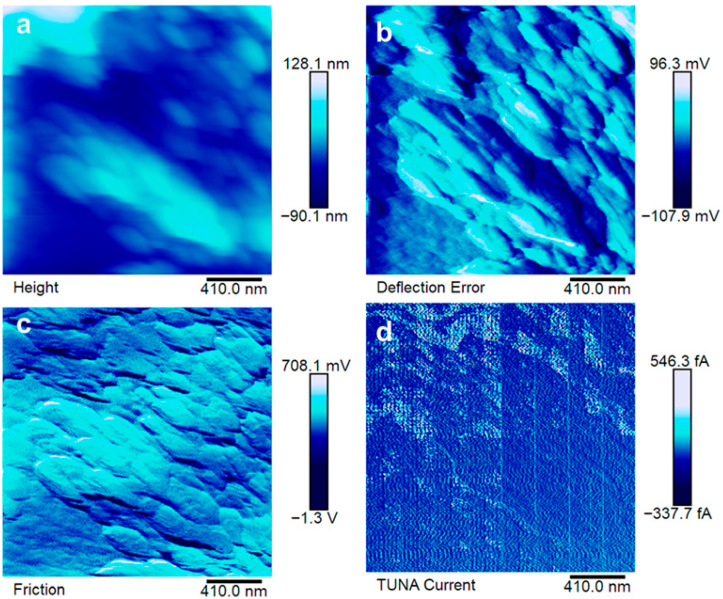
Atomic force microscopy images of EP7HTM: height image (**a**), deflection error image (**b**), friction image (**c**), TUNA current (**d**).

The morphological features of the produced samples have been characterized via SEM and TUNA microscopies to determine the distribution of EG particles in the epoxy nanocomposite. The results of this analysis are reported in Figure 7, Figure 8, Figure 9 and Figure 10.

Figure 7a–d show that the filler distribution is sensitively affected by the dispersion method. In particular, for the same load of nanofiller, the centrifugal planetary mixing seems to cause a more effective distribution (see Figure 7d–f). This more effective distribution occurs through a reduction in the EG aggregate dimensions. The different dispersion states result in substantial differences in electrical behavior and water transport properties, as discussed in the following sections. For example, regarding electrical conductivity, it is expected that the centrifugal planetary makes it challenging to form conductive electrical paths (see sample d), which instead favored sample e, where the filler was dispersed through ultrasonication. It can be seen to be even more difficult in the case of sample f, where the EG aggregates are more delimited by large zones of the insulating epoxy matrix. These results are further confirmed by the image processing analysis reported in Table S3 of the Supplementary Materials.

The electrical paths have been investigated at the sub-micrometric level via TUNA. The results are reported in Figure 8, Figure 9 and Figure 10.

The good distribution of EG in EP7CEN is confirmed by the analysis reported in Figure 8a–c. The electrical current values detected during the TUNA analysis (up to 813.1 fA) enable the electrical recognition of EG presence in the epoxy matrix from a morphological perspective. Comparing these results with EP7SON (see Figure 9), it is evident that the current values that flow in this last sample (up to 3.4 pA) are higher than the others, suggesting the formation of highly conductive electrical paths thanks to the ultrasonication mixing, in accordance with the SEM image (see Figure 7). Lastly, for EP7HTM, it is possible to recognize lower electrical current values (up to 546.3 fA), which suggest a weaker formation of the electrically conductive network (probably, because of the formation of clusters reported in Figure 7).

### 3.5. Electrical Analysis

**Figure 11:**
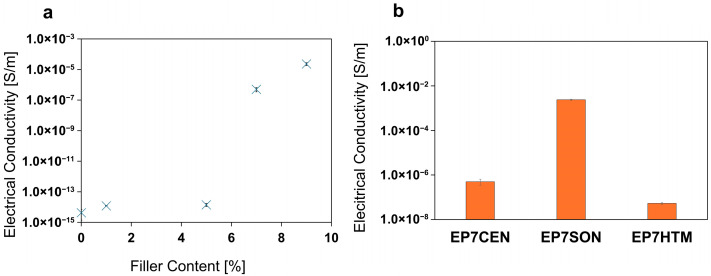
Electrical conductivities of the epoxy nanocomposites (EPXCEN) at various filler content (**a**). Influence of the mixing method on the electrical conductivity of the sample (**b**).

The direct current (DC) electrical conductivity of the obtained samples has been characterized in bulk. The results are reported in Figure 11. In particular, Figure 11a shows the EPT curve of the sample containing EG embedded in the polymeric matrix through the centrifugal planetary mixing.

In accordance with the morphological analysis reported in Section 3.4, the increase in the filler content leads to a sharp increase in the electrical conductivity of the formulation around 7% of filler content (see Figure 11a). However, it is worth noting that the range of filler content corresponding to the increase in electrical conductivity of the nanocomposite is strictly related to the formation of the EG pathways, depending on the mixing methods. To better understand this aspect, Figure 11b presents the electrical conductivity values of the samples with a 7% filler content, obtained using the three different mixing methods analyzed. The results presented in Figure 11 strictly agree with the TUNA results (see Figure 8, Figure 9 and Figure 10). The arrangement of the EG particles in the matrix is strongly affected by the mixing method used, as reported in Section 3.4 (see Figure 7). As previously highlighted, EP7HTM morphologically displays the presence of clusters (due to a non-homogeneous distribution of the filler) and, consequently, there are broad zones with poor electrical contacts that cause an increase in the overall resistivity of the sample.

On the other hand, ultrasonication ensures the most efficient formation of electrical contacts in the sample, resulting in the formation of an efficient continuous network of conductive particles, which leads to the development of composites that display the highest electrical conductivity among the various samples loaded with 7% EG. Even if EP7CEN also leads to a good distribution of EG in the epoxy matrix (see Figure 7), the graphitic particles still do not tend to form continuous paths for the electrical current. For this reason, it shows lower electrical conductivity than EP7SON. Moreover, it should be considered that the increase in electrical conductivity also indicates the formation of paths that allow heat transfer, not only through a phononic mechanism, but also through an electronic mechanism [46,47]. This phenomenon explains why the curing degree of the three types of samples differs, being higher in EP7SON and lower in EP7HTM. The heat transfer during curing in the oven is more efficient in the EP7SON sample, resulting in higher values of the curing degree in the final sample.

### 3.6. Water Sorption Analysis

The difference in water sorption of nanocomposite epoxy samples prepared using different mixing methods has been evaluated and compared to that of the unfilled EP.

**Figure 12:**
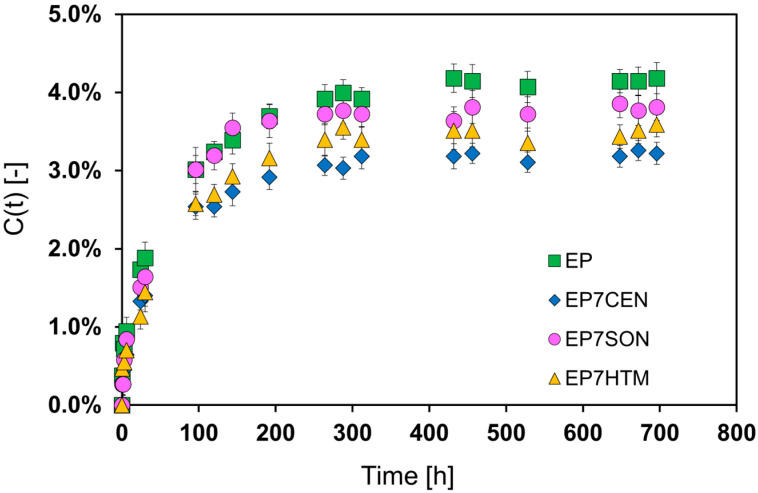
Water sorption of nanocomposite samples.

The water sorption was evaluated following the procedure described in Section S1 of Supplementary Materials. The results are reported below in Figure 12.

The sorption value at equilibrium (C_eq_) is 4.18% for EP. However, by incorporating nanofillers into the epoxy resin, the C_eq_ decreases for all the mixing methods investigated. However, it is worth noting that the use of different mixing methods leads to different values of C_eq_, which range from 3.81% for EP7SON to 3.22% for EP7CEN. Hence, a different distribution of EG particles within the epoxy matrix results in a different performance in the barrier properties. As shown in Figure 8, the centrifugal mixing ensures a good distribution of EG within the epoxy matrix. In fact, for this mixing method, a 23% reduction in the equilibrium concentration of water (C_eq_) is detected. This reduction entity is quite substantial and highly desired for epoxy composites designed for use in the transport sector, particularly in the aeronautical industry.

The most efficient decrease in water C_eq_ is detected for the EP7CEN, whereas the least efficient method appears to be ultrasonication, for which a slight reduction in water C_eq_ can be detected only in the plateau condition of the curves.

The Fickian diffusion model is extensively applied for the description of the water sorption mechanism into epoxy resins [48]. In the present research, the Fick model has been applied to determine the diffusivity (D) values of the various systems [49]. Since the inclusion of graphite increases the tortuosity of the water molecules’ path, a reduction in the diffusivity of the system is observed for all samples loaded with EG. The diffusivity values are reported below in Table 3.

According to the morphological analysis in Figure 7, centrifugal planetary mixing ensures a uniform distribution of EG in the epoxy matrix. This phenomenon leads to an increase in the tortuosity of the path, which leads to a decrease in the diffusivity [50]. However, different mixing methods lead to different decreases in the diffusivity, because of the different distribution of the EG in the matrix (see Figure 7). In fact, the system EP7HTM, which shows the presence of clusters (and consequently leads to the formation of zones with low path tortuosity), presents a low decrease in diffusivity. On the other hand, EP7SON, and mostly EP7CEN, display the lowest diffusivity values thanks to the more homogeneous distribution of expanded graphite in the matrix.

Water molecules can be considered as a molecular “probe”, indicating that a significant fraction of the resin can interact with the water molecules, as can the EP unfilled matrix. The arrangement of the graphitic layers in the matrix, in the case of the centrifugal planetary mixing, allows for preventing the access of water to a significant fraction of the matrix. As confirmed by the FT-IR analysis in Figure 6, the epoxy matrix well interacts via hydrogen bonding with the EG (see Section 3.3). Due to these hydrogen bondings, the interactions of water with the matrix are hindered, resulting in a decrease in the C_eq_ for the EP7CEN.

## 4. Conclusions

The dispersion methods of fillers into epoxy matrices sensitively affect the final properties of the materials. In the present research, different mixing methods have been taken into account for the dispersion of the expanded graphite: centrifugal planetary mixing, ultrasonication, and high-temperature mixing. Expanded graphite is distributed differently by varying the mixing methods, leading to relevant differences in the curing degree of the sample, and mostly in the thermomechanical properties, with the glass transition temperature that varies from 189 °C for EP7HTM to 212 °C for EP7SON. This is probably due to the most efficient heat transfer in EP7SON, thanks to the formation of a continuous EG network (confirmed via SEM and TUNA analysis). The different distribution of EG in the epoxy matrix leads to variations in the electrical conductivity of several orders of magnitude (from 10^−8^ S/m for EP7HTM to 10^−3^ S/m for EP7SON), even by using the same EG percentage. The effect of the mixing methods on the barrier properties of the epoxy resins has been investigated. The behavior of the water sorption curves of the analyzed samples highlights an impressive correlation with the structural and morphological features of the samples, providing relevant information on the nanofiller dispersion in the hosting epoxy matrix. The presence of expanded graphite in the epoxy matrix always results in a decrease in the equilibrium concentration of water (C_eq_), regardless of the graphite dispersion method employed. The extent of the reduction, however, depends on the dispersion method. The more efficient decrease in water C_eq_ is detected for the sample in which centrifugal planetary mixing was used to disperse EG nanoparticles in the matrix. The less efficient method appears to be ultrasonication, for which a slight reduction in water C_eq_ can be detected only in the plateau condition of the curves. If we consider the water molecule as a molecular “probe”, we can deduce that a significant fraction of the resin is very similar to the EP unfilled matrix. 

The arrangement of the graphitic layers in the matrix, in the case of the centrifugal planetary mixing, allows for preventing the access of water to a significant fraction of the matrix. The result seems to be confirmed by TUNA and SEM results. A comparison of TUNA images of the sample obtained through EG centrifugal planetary mixing and that of the sample obtained through EG ultrasonication highlights that the ultrasonication determines arrangements of extended blocks of graphite along the sample, leaving big zones of the electrically insulating matrix. The observation of images corresponding to SEM images of the EP7CEN and EP7SON samples, respectively, fully supports this interpretation.

## Figures and Tables

**Table 1:** Produced samples.

Sample	EG Content [wt. %]	Mixing Method
EP	0	/
EP1CEN	1	Centrifugal Planetary Mixing
EP3CEN	3	Centrifugal Planetary Mixing
EP5CEN	5	Centrifugal Planetary Mixing
EP7CEN	7	Centrifugal Planetary Mixing
EP7SON	7	Ultrasonication
EP7HTM	7	High Temperature Mixing
EP9CEN	9	Centrifugal Planetary Mixing

**Table 2:** FT-IR signals assignment for EP sample.

Assignment	Wavenumber (cm^−1^)		Reference
	Uncured	Cured	
ν O-H	-	3426	[39]
ν N-H	3467, 3370	-	[23,39]
δ N-H	3240, 1630	-	[23,39]
ν aliphatic C-H	2968, 2927, 2875	2968, 2927, 2875	[40]
ν C=C	1596, 1508, 1452	1596, 1508, 1452	[40]
ν alkyl-aryl ethers C-O-C	1240, 1036	1240, 1036	[40,41]
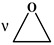	915	-	[39,40]

ν: stretching vibration; δ: bending vibration.

**Table 3:** Diffusivity parameter of different epoxy systems.

	EP	EP7CEN	EP7SON	EP7HTM
D [mm^2^/s]	9.83 × 10^−6^	3.42 × 10^−6^	5.39 × 10^−6^	5.90 × 10^−6^
R^2^	0.9978	0.9992	0.9994	0.9985

## Data Availability

The original contributions presented in this study are included in the article/Supplementary Materials. Further inquiries can be directed to the corresponding author.

## Supplementary Materials

The following supporting information can be downloaded at https://www.mdpi.com/article/10.3390/polym17202759/s1, Figure S1: SEM images and binary conversion of EP7CEN (a1, a2), EP7SON (b1, b2), and EP7HTM (c1, c2); Figure S2: DTGA of EP samples unloaded and loaded with 7% of EG in air (a) and N_2_ (b); Figure S3: Curing degree evolution during dynamic DSC at 10 °C/min (a) and during isothermal DSC at 160 °C; Figure S4: XRD spectra (a) and analysis of the number of layers for EG (b); Table S1: Thermal degradation parameters of unfilled and filled epoxy resin; Table S2: R^2^ values; Table S3: Area covered by EG on the composite surfaces.
