# Supplementary material for: Effects of the Mixing Method of Expanded Graphite on Thermal, Electrical, and Water Transport Properties of Thermosetting Nanocomposites
Source: Polymers (Basel). 2025 Oct 15;17(20):2759. doi: 10.3390/polym17202759 (PMC12566862; doi:10.3390/polym17202759)
Supplement: Supplementary file 1 [file polymers-17-02759-s001.zip › polymers-3899084-supplementary.pdf]

polymers

MDPI

Supplementary Materials

Effects of the Mixing Method of Expanded Graphite on Thermal, Electrical, and Water Transport Properties of Thermosetting Nanocomposites

Raffaele Longo [1,*], Elisa Calabrese [1], Francesca Aliberti [1], Luigi Vertuccio [2], Giorgia De Piano [1], Roberto Pantani [1], Marialuigia Raimondo [1] and Liberata Guadagno [1,*]

[1] Department of Industrial Engineering, University of Salerno, Via Giovanni Paolo II, 132, 84084 Fisciano, Italy; elicalabrese@unisa.it (E.C.); faliberti@unisa.it (F.A.); gdepiano@unisa.it (G.D.P.); rpantani@unisa.it (R.P.); mraimondo@unisa.it (M.R.)

[2] Department of Engineering, University of Campania "Luigi Vanvitelli", Via Roma 29, 81031 Aversa, Italy; luigi.vertuccio@unicampania.it

* Correspondence: rlongo@unisa.it (R.L.); lguadagno@unisa.it (L.G.)

S1. Epoxy Resin Characterization

Thermogravimetric analyses (TGA) were performed on the samples by using a Mettler Toledo Mettler TC-10 (Columbus, Ohio, US) thermobalance in air and N_2 atmosphere (50 mL × min^{-1}) with 10 °C × min^{-1} heating rate from 30 to 900 °C.

Differential Scanning Calorimetry (DSC) dynamic analyses were performed using a Mettler Toledo DSC 822e (Columbus, Ohio, US) under N_2 flow (50 ml × min^{-1}) with a 10 °C/min heating rate to determine the curing degree of the epoxy resin. The area below the enthalpic curves was used to evaluate the curing degree (C.D.) using Equation (S1):

$$C.D. = \frac{(\Delta H_{Dyn} - \Delta H_{Residual})}{\Delta H_{Dyn}} * 100 \quad \text{(S1)}$$

where ΔH_{Dyn} is the total heat of the reaction, and $\Delta H_{Residual}$ is the residual heat of the sample obtained after the curing cycle.

Fourier Transform Infrared (FT-IR) spectroscopic analyses were carried out on the epoxy resin-based samples and on exfoliated graphite. FT-IR spectra were recorded in absorbance using a Bruker Vertex 70 FT-IR spectrophotometer with a resolution of 4 cm^{-1} (32 scans collected) in the range 4000–400 cm^{-1}. Regarding the nano-filler (EG) and the cured samples (EP, EP7SON, EP7HTM, and EP7CEN), to obtain the spectra, their powders were dispersed in KBr pellets, which served as carriers. Concerning the epoxy sample EP, the FT-IR investigation was also carried out on the uncured epoxy mixture (the mixture before the polymerization reaction), and the spectrum was recorded by spreading a thin layer of the viscous liquid mixture on a KBr pellet.

Wide-angle X-ray diffraction (XRD) patterns were obtained using an automatic Bruker D8 Advanced diffractometer (Billerica, Massachusetts, US), in reflection, at 35 KV and 40 mA, using nickel-filtered Cu Kα radiation (1.5418 Å).

Electrical characterization was necessary to choose the amount of filler most suitable for imparting the desired functionality, performing 2-probe measurements for the sample above 7% in weight of EG content. It was performed by using a Keysight 3458a digital multimeter to monitor the current and applying voltage by using an external power supply (Elektro Automatik, EA-PSI 9500-10 T DC Power Supply, Helmholtzstraße, Viersen, Germany). Below 7%EG, the epoxy mixtures have been measured using Keysight b2980.

The DC volume conductivity measurements of the composites have been performed by using disk-shaped specimens of about 2 mm in thickness and 50 mm in diameter. Before performing the electrical measurements, the samples are thermally pre-treated at 90 °C for 24 h to remove any traces of moisture or solvents. Then, both sides of the samples have been metallized (circular form of about 22 mm in diameter) with silver paint (Alpha Silver Coated Copper Compound Screening, with a resistivity of 0.7 Ω-square) to reduce the effects of surface roughness and to ensure Ohmic contacts.

SEM was used to perform morphological analysis using the Phenom ProX microscope. The samples were metalized using an Agar Auto Sputter Coater (Parsonage Ln, Stansted CM24 8GF, UK) with a thin gold layer of 25 nm.

For the image processing of the SEM images, MATLAB Software has been used. Firstly, the SEM images are converted into binary matrices. Since the graphite is generally slightly different from the surrounding matrix, it can be easily recognized during the image processing (see image below). The total white pixels (corresponding to the EG) and dark pixels (corresponding to the matrix) for each image can be evaluated.

The SEM images converted into binary are reported in **Figure S1**.

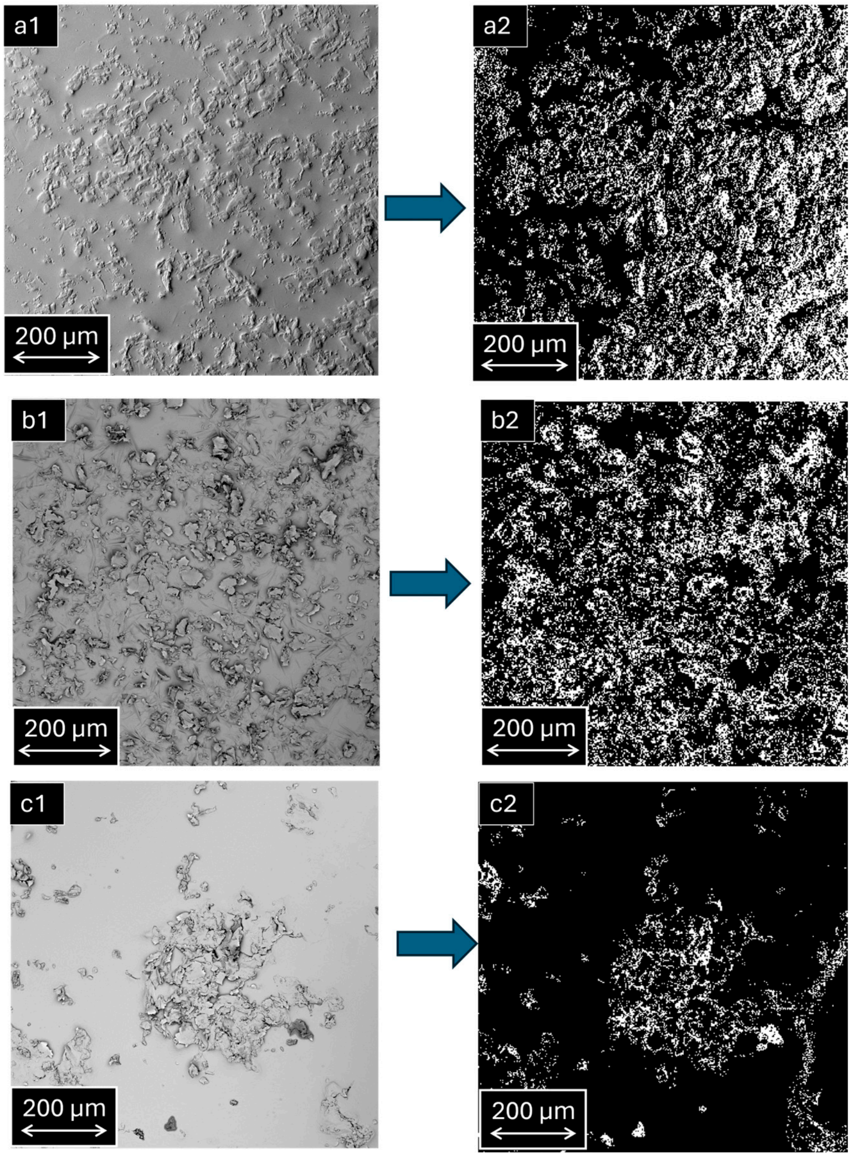

Figure S1. SEM images and binary conversion of EP7CEN (a1, a2), EP7SON (b1, b2) and EP7HTM (c1, c2).

In this way, by dividing the number of white pixels by the total number of pixels of the image, it is possible to determine the surface of the samples that is covered by EG.

The conductivity map of the nanodomains of the etched samples was carried out by TUNA operating in contact mode with a cantilever holder and the sample containing conductive expanded graphite, electrically connected to an external voltage source. Platinum-coated probes with nominal spring constants of 35 N m^{-1} and an electrically conductive tip of 20 nm were used to perform the TUNA measurements, adopting the following control parameters: DC sample bias from 1 V to 2 V, current sensitivity of 1 pA/V, current range of 200 nA, number of pixels in X and Y (samples/lines) equal to 512, 0.5 Hz scan rate, 2.000 integral gain and 5.000 proportional gain. In contact mode, the tip is in uninterrupted contact with the sample. To ensure the reproducibility of the measurements, various areas of the samples were analyzed. The pictures were examined using the Bruker software Nanoscope Analysis 1.80 (Build R1.126200). The lateral resolution of the TUNA pictures is approximately equal to the end of the tip radius (20 nm). Maintaining a constant force between the tip and the sample makes it possible to detect topographic and current images simultaneously.

The epoxy resin samples have been cut to similar dimensions so that all the samples weigh 0.22 g and 0.27 g. The thickness of the water absorption test samples was made small, compared to their width and length. After conditioning samples at 120 °C under vacuum for 24 h to ensure complete dryness, the specimens were placed into distilled water chambers in thermostated baths maintained at a constant temperature of 25 °C (water activity = 1). Three tests for each type of sample have been performed. This methodology has been selected after different mechanical tests, which have proven that the drastic treatment at 120 °C under vacuum for 24 h is able to remove all the water in the resin. This also causes the disappearance of the peak related to the presence of bound water in the dynamic mechanical spectrum. The specimens were weighed periodically using a digital balance with 0.1 mg resolution to determine the percent weight change and, thus, water uptake. The water gain percentage, Ct%, was determined from Equation (S2):

$$C(t) = \frac{W(t) - W_0}{W_0} * 100 \quad \text{(S2)}$$

In Equation (S2), W(t) is the weight of the water-sorbed epoxy specimen at time t, and W_0 is the initial weight of the dry specimen. The equilibrium concentration of water C_{eq} was calculated considering the maximum amount of absorbed water in the plateau condition. The specimens were periodically removed, dried, and immediately weighed, and then returned to the water bath. The step of drying is performed to ensure the removal of excessive surface (superficial) water. The specimens were gently wiped dry with clean, lint-free tissue paper. The described absorption procedure yielded a series of water gain versus time curves.

The Fickian diffusive model has been applied to evaluate the diffusivity (D) by modeling the water sorption curves according to Equation (S3).

$$\frac{W(t)}{W_\infty} = 1 - \frac{8}{\pi^2} * \sum_{n=0}^{\infty} \frac{exp\left(-D*(2n+1)^2*\pi^2*\frac{t}{L^2}\right)}{(2n+1)^2} \quad \text{(S3)}$$

Where t is time, L is the thickness and W_∞ is the plateau weight of the epoxy samples.

S2. TGA Analysis

Derivative Thermogravimetric Analysis (DTGA) of the unloaded epoxy resin (EP) and composite resin filled with 7%EG (EP7CEN, EP7SON and EP7HTM) are reported in **Figure S2**.

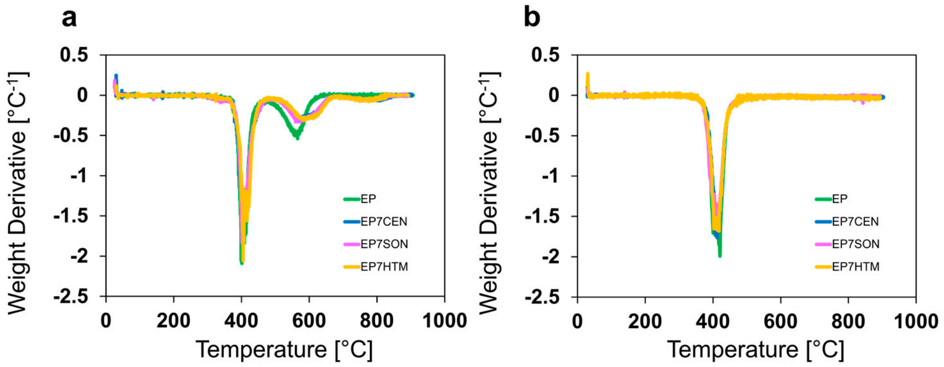

Figure S2. DTGA of EP samples unloaded and loaded with 7% of EG in air (a) and N_2 (b).

By the analysis of the curves in **Figure 1**, the temperatures of the 5 wt% weight loss ($T_{5\%}$) and the 50 wt% weight loss ($T_{50\%}$) have been evaluated for all the different systems. The results are reported below in **Table S1**.

Table S1. Thermal degradation parameters of unfilled and filled epoxy resin.

		EP	EP1CEN	EP3CEN	EP5CEN	EP7CEN	EP7SON	EP7HTM	EP9CEN
$T_{5\%}$ [°C]	N_2	386.8	387.6	386.4	368.4	384.7	381.2	384.3	387.7
	Air	385.0	387.9	387.2	386.9	384.9	376.7	381.3	387.7
$T_{50\%}$ [°C]	N_2	418.5	419.3	420.9	420.4	422.4	420.5	421.4	424.0
	Air	419.7	423.1	423.1	429.0	428.7	428.8	425.9	431.7

S3. Kinetic Analysis

Kinetic analysis of the curing degree evolution over time for various EG content has been performed both in dynamic and in isothermal DSC tests, comparing the evolution of the curing degree of the EP sample and the sample of EP loaded with 7%EG (EP7CEN).

The curing degree for the isothermal DSC has been evaluated in accordance with literature as reported in Equation S4.

$$C.D. = \frac{1}{\Delta H_{tot}} * \int_0^t \left(\frac{dH}{dt}\right) dt \quad \text{(S4)}$$

By the comparison of the curing degree in dynamic and isothermal conditions, it is evident that the inclusion of the EG into the epoxy matrix causes a decrease in the curing kinetics, probably because of the decreased mobility of the chains. The results are reported in **Figure S3**.

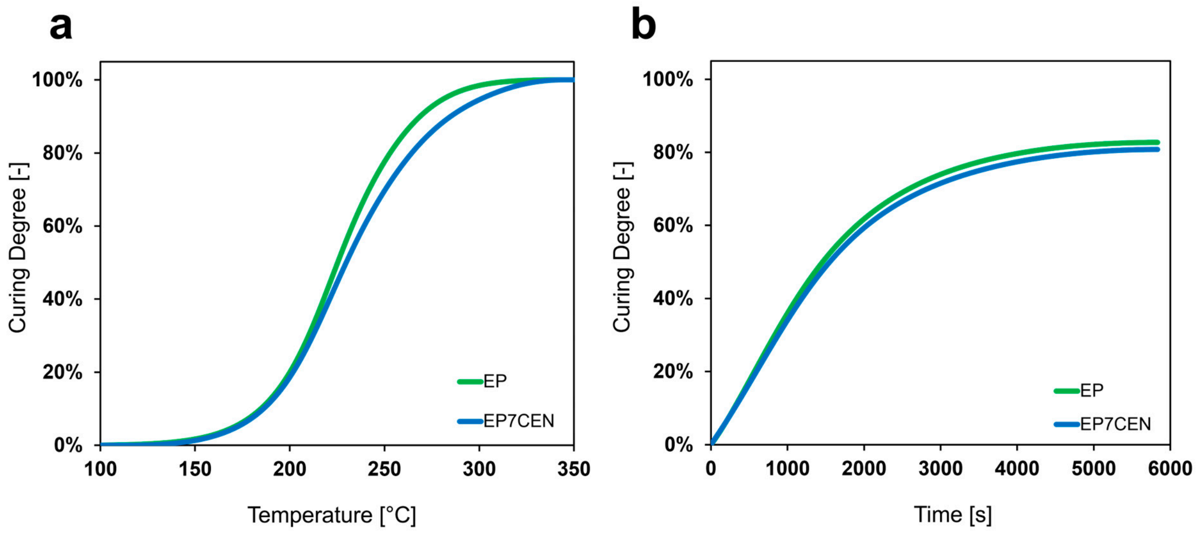

Figure S3. Curing degree evolution during dynamic DSC at 10 °C/min (a) and during isothermal DSC at 160 °C.

S4. FT-IR of Epoxy Nanocomposites

The reliability of the deconvolution procedure's results is confirmed by the extremely high values of the square of the correlation coefficient (R^2), which are reported in **Table S2**.

Table S2. R^2 values

Sample	R^2
EP	0.9995
EP7SON	0.9996
EP7HTM	0.9990
EP7CEN	0.9991

S5. XRD Analysis of Epoxy Nanocomposites

XRD analyses have been performed to investigate how the dispersion method affects the structure of the nanofiller. The interlayer distance and the number of layers of the EG particles included in the epoxy matrix have been evaluated and compared to the EG before-the inclusion in the polymeric matrix.

The d-spacing of the EG in the epoxy matrix was evaluated for nanocomposite samples obtained with various mixing methods by applying Bragg's Law, reported in Equation (S5):

$$n * \lambda = 2 * d * \sin(\theta) \qquad \text{(S5)}$$

where n is an integer (in this case, n=1), λ is the radiation source wavelength (1.54184 Å), d is the interlayer distance, and θ is the diffraction angle at which the peak is observed.

On the same spectra, the authors evaluated the average dimensions of the EG crystallites by applying Scherrer's Equation, reported in Equation (S6):

$$D = \frac{K*\lambda}{\beta*\cos(\theta)} \quad (S6)$$

Where K is Scherrer's constant (in this case, K=0.9), β is the full width at half maximum of the peak. In this way, taking as reference the main peak of EG (002), the number of layers of EG was evaluated in accordance to Equation (S7).

$$Number\ of\ layers = \frac{D_{002}}{d_{002}} \quad (S7)$$

The XRD spectra and evaluation of the number of layers are reported below in **Figure S4**.

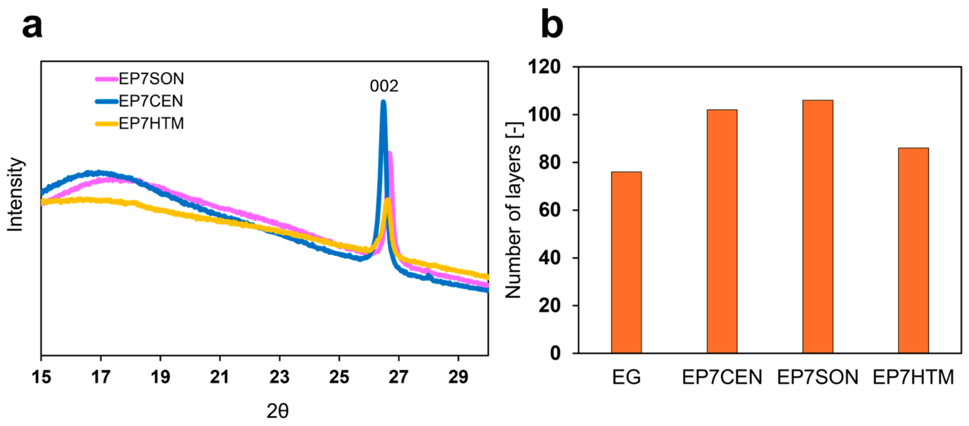

Figure S4. XRD spectra (a) and analysis of the number of layers for EG (b).

From the analysis of the spectra, it is possible to recognize the amorphous halo of EP in all nanocomposite systems. However, in the nanocomposite systems, a sharp peak at around 26.5° (002), characteristic of the expanded graphite, is detectable.

By observing **Figure S4b**, it is possible to observe that the number of layers of EG increases because of the mixing. This indicates that all the mixing analyzed methods slightly favor the assembling of graphene layers of the EG. This means that results commented and discussed in the main text must be attributed only to a different distribution of the graphitic block in the hosting epoxy matrix.

S6. Image Processing of SEM

By dividing the number of white pixels by the total number of pixels of the image, it is possible to determine the surface of the samples that is covered by EG. In particular, the results are reported below, which display a better distribution of EG for EP7SON and EP7CEN compared to EP7HTM. The results are reported in **Table S3**.

Table S3. Area covered by EG on the composite surfaces.

	**Area Covered by EG [%]**
EP7CEN	27.7
EP7SON	26.5
EP7HTM	6.22
